# Modelling photosynthetic responses by day and night during initial water stress in *Pulmonaria vallarsae*

**DOI:** 10.1111/ppl.70004

**Published:** 2024-12-10

**Authors:** Paolo Pupillo, Francesca Sparla, Bruno Andrea Melandri, Paolo Trost

**Affiliations:** ^1^ Department of Pharmacy and Biotechnology University of Bologna Bologna Italy

## Abstract

The relationships between photosynthesis and initial water deficit stress were investigated by chlorophyll fluorescence analysis in *Pulmonaria vallarsae*, a shade tolerant, perennial C3 herb by following changes of light response curves (LRCs) in single leaves during water shortage. We devised an LRC model based on two interacting rectangular hyperbolae (DH model) for the low (*H*1) and the high irradiance regions (*H*2), characterized by two parameters: maximum extrapolated ETR (*V*1, *V*2) and half‐saturation irradiance (*K*1, *K*2). While *H*1 is assumed to represent an ETR‐related function, *H*2 may reflect Rubisco activity. Plants were subjected to four days of water restriction in summer and tested every 12 h. While daytime values remained relatively stable, increasing water stress gradually induced a night depression of photosynthesis mainly dependent on decreasing ETR with concomitant reduction of PSII‐dependent parameters (Φ_PSII_) and fluorescence‐related functions, while nonphotochemical quenching (NPQ) was strongly enhanced. In terms of the DH model, the night depression of photosynthesis featured a night drop of *V*2 and *K*2 followed by decreases of *V*1 and *K*1. The *H*2 hyperbola was more stress responsive than *H*1 and frequently showed a reversible decrease of nocturnal *H*2 parameters (bright illumination constraint, BIC). *Pulmonaria* plants tested during winter with very low water stress displayed LRCs resembling rectangular hyperbolae, similar during day and night. The DH model is shown to yield accurate and perspicuous photosynthetic parameters representing the principal components of an LRC and to be well suitable to document the day/night divergence of photosynthetic capacity during a water deficit stress.

## 1 INTRODUCTION

The circadian control of photosynthesis and related processes in plants has been known for decades, since early reports on daily oscillations of photosynthetic activity (Pallas et al., 1974; Kerr et al., 1985; Hennessey & Field, 1991), stomatal regulation (Martin & Meidner, 1971; Holmes & Klein, 1986), turnover of antenna proteins (Millar et al., 1992) and of carbon cycle enzymes (Martino‐Catt & Ort 1992). The rhythm is currently recognized to regulate most chloroplast processes (Dodd et al., 2014) with entrainment by photosynthesis products (Haydon et al., 2013; Philippou et al., 2019) and blue light (Litthauer et al., 2015; de Barros Dantas et al., 2023). In *Pulmonaria vallarsae*, a shade‐tolerant herb of Italian uplands, the circadian rhythm drives a night reduction of potential photosynthesis capacity and chlorophyll fluorescence emission, with the enhancement of nonphotochemical quenching (Pupillo et al., 2022). This condition was defined as the night depression of photosynthesis and appeared to be associated with the experience of water shortage in *P. vallarsae*. That circadian rhythm‐dependent stomatal regulation, too, was associated with water deficit has long been known (Raschke, 1975; Mansfield et al., 1990; Lawson et al., 2010). Many plant functions besides those connected to photosynthesis are controlled by rhythm (Millar, 2016) and some may also be related to drought. For example, water stress induced a circadian oscillation of enhanced water transport and night growth in some species (Caldeira et al., 2014).

Therefore, it was of interest to investigate the development of the night depression of photosynthesis during a water shortage period, the more so as the issue is related to a long‐debated question concerning plant photosynthetic capacity during drought. According to one view, water deficit stress directly affects the photosynthetic metabolism (Tezara et al., 2002; Zivcak et al., 2013) and it is now clear, that severe drought impacts both photosystems (Hu et al., 2023; Sapeta et al., 2023). On the other hand, the decline of (diurnal) photosynthetic activity during a moderate drought has been entirely attributed to stomatal regulation (Mansfield et al., 1990; Bota et al., 2004) and was counteracted by saturating CO_2_ levels (Cornic & Briantais, 1991; Quick et al., 1992; Chaves et al., 2009), although the point was controversial (Björkman & Powles, 1984; Graan & Boyer, 1990; Martin & Ruiz‐Torres, 1992). Since the effect of water stress on nocturnal photosynthetic capacity has rarely been probed, the day/night dimension of drought‐limited photosynthesis also deserves consideration.

We have addressed these problems through an investigation of *P. vallarsae* adaptation to a mild water deficit by using pulse amplitude modulated (PAM) fluorometry tests to obtain photosynthetic LRCs during the day and night. Reading and interpreting the form of an LRC was a critical step. A very early model of the LRC (Blackman, 1905) envisioned a linear increase of photosynthesis with increasing light energy, and sharply reaching a maximum when the CO_2_ supply became limiting. This plausible but schematic approach inspired most later studies, though it fails to reflect the complex biology underlying an LRC, which Maskell (1928) therefore redesigned as a rectangular hyperbola by analogy with an enzymatic saturation curve. Since then, models based on non‐rectangular hyperbolae with a curvature term have gained extensive use (Marshall & Biscoe, 1980; Farquhar et al., 1980; Thornley 1998; Xu et al., 2014; Herrmann et al., 2020). An alternative approach based on Chl excitation properties adopts a non‐asymptotic function for the LRC, predicting a decline of photosynthesis at elevated irradiance (Ye et al., 2013, 2024). It seems clear that photosynthetic assimilation at high illumination is often limited by CO_2_ availability, the extent of photorespiration, and/or Rubisco activation state (von Caemmerer, 2000; Bernacchi et al., 2003; Salvucci & Crafts‐Brandner, 2004; Jiang & Rodermel, 2009; Yamori et al., 2012). The low‐irradiance (LI) domain of an LRC allows the calculation of maximum quantum yield and potential rates of photosynthetic electron transport and RuBP regeneration, which are commonly down‐regulated to match the photosynthetic capacity of the Rubisco‐limited high‐irradiance (HI) domain (Weis & Berry 1987; Ögren, 1993). Why then, not treat the two major domains of the LRC as distinct albeit interacting units, liable to be separately examined in some situations? Starting from this idea, we have developed a new approach based on the apposition of rectangular‐hyperbolic equations for both regions of an LRC, i.e. LI (hyperbola 1, *H*1) and HI (hyperbola 2, *H*2), hence defined the double‐hyperbola (DH) model. The DH model when compared to other approaches is shown to adequately fit real photosynthetic responses of *P. vallarsae* in the irradiance range of interest and to be able to catch subtle changes of rate and light dependence of the major components of an LRC. Using this method, we have investigated the progress of photosynthetic adaptation during a few days of water shortage, with new evidence on the origin of the circadian night depression of photosynthesis. We also confirmed the absence of a night depression of photosynthesis during winter months in *Pulmonaria* leaves, that showed similar day/night LRC patterns approaching rectangular hyperbolae. These findings highlight the strict relationship of the circadian oscillations of photosynthesis with water deficit stress, as well as the role of stress in the structure of light response curves.

## 2 MATERIALS AND METHODS

### 2.1 Plant growth

Most experiments were carried out in late spring and summer with plants of *Pulmonaria vallarsae* Kerner subsp. *apennina* Cecchi et Selvi (Italian lungwort, family Boraginaceae), a perennial understory herb of Italian uplands, relatively shade tolerant, with two basal leaf generations in a year plus a late winter flowering stage. Plants were collected on hills near the city of Bologna (Colle di Paderno, ca. 300 m altitude) and grown in pots in a greenhouse in the Orto Botanico of Bologna University as described in Pupillo et al., (2022) with evening irrigation every two days. Mature, basal rosette leaves of *P. vallarsae* are large and sturdy enough to stand repeated manipulations unharmed, so a single leaf per plant was always used during serial tests. Water deprivation experiments were started with plants irrigated the night before, at night temperatures of 18‐22°C and maximum daytime temperatures of 29‐30°C, with Chl fluorescence tests usually at about 10 am and 11 pm each day (hence eight tests: *Dd*1, *Dn*1; *Dd*2, *Dn2; Dd*3, *Dn*3; *Dd*4, *Dn*4). For comparison, experiments were also performed using outdoor growing plants of the Orto Botanico during suitable autumn and winter periods, each test being preceded by one hour's plant acclimation at 22°C.

### 2.2 Analysis of chlorophyll fluorescence

Chl *a* slow fluorescence was assayed with the Imaging PAM 2000 fluorometer (Walz GmbH) using the pulse amplitude modulated (PAM) technology and associated PIM^R^ software via a dedicated computer (Recchia et al., 2017). One area of interest of 3 mm diameter was chosen on the lower (abaxial) surface of the leaf under study, which was held in place by a horizontal clip. For fluorescence‐related parameters, we shall follow the nomenclature of Maxwell and Johnson (2000). Minimum fluorescence (*F*_o_) was obtained by applying measuring light pulses (1 ms) of low frequency (1 Hz). Maximum fluorescence (*F*°_m_) was determined before each test through a 0.8 s pulse of 2.4 mmol photons m^‐^^2^ s^‐^^1^ (saturation flash). *F’*_m_ is the maximum fluorescence measured with a saturating pulse during illumination, *F*_t_ . is instant fluorescence. The effective quantum yield of PSII photochemistry is Φ_PSII_ = (*F’*_m_ – *F*_t_)/*F’*_m_; nonphotochemical quenching NPQ = (*F*°_m_‐*F’*_m_)/*F’*_m_; photochemical quenching qP = (*F’*_m_ – *F*_t_)/(*F’*_m_ – *F’*_o_); photosynthetic electron transport rate ETR = Φ_PSII_ x PPFD x 0.5 x Abs, assuming equal distribution of light energy between both photosystems (Björkman & Demmig, 1987). Absorptivity is determined by the instrument (Abs = 1‐R/NIR).

### 2.3 PAM fluorometer tests and data processing

PAM fluorometer tests were performed in the laboratory during the late spring and summer months of years 2021 to 2023. Prior to each test, light‐exposed plants were held in the dark for 10‐15 min. A standard test was started by a blue saturation pulse, followed by a 5‐min light induction pretreatment of blue actinic light (450 nm peak, 100 μmol photons m^‐^^2^ s^‐^^1^) with 15 saturation pulses released at 20 s intervals. Flash‐induced fluorescence peaks (FIP = *F’*_m_ – *F*_t_) ranging between 4% and 45% of *F*°_m_ were thus triggered, and the last FIP of the series was used as an informative parameter of the current activation state of photosystem II. The light induction pretreatment was followed by a light response curve (LRC) stimulated by 13 blue actinic light steps of increasing PPFD (1 to 460 μmol photons m^‐^^2^ s^‐^^1^) of 20 s each with a final saturation pulse, thereby inducing Chl fluorescence spikes (*F*’_m_) and consequent changes of fluorescence‐related parameters Φ_PSII_, qP, NPQ, and ETR (calculated by software). Although LRCs derived from Chl fluorescence may represent trends of any of the above parameters and others, the term LRC is commonly referred to photosynthetic electron transport alone, hence ETR‐LRC in this paper. ETR‐LRC data were analysed by nonlinear regression (CoHort software) using different models: (i) the function of the Non Rectangular Hyperbola (NRH) model (Farquhar et al., 1980) yielding parameters J_max_, Φ, and θ (where J_max_ is the light saturated rate of linear electron transport, Φ is the maximum quantum yield of linear electron transport, and θ is a curvature factor); (ii) the non‐asymptotic function of Ye et al., (2013) based on parameters α, β and γ (where α is maximum quantum yield of linear electron transport, β is an LRC rate decline term and γ is a light saturated coefficient); and (iii) the double‐hyperbola or DH model developed in this paper.

### 2.4 The double‐hyperbola (DH) model

The double‐hyperbola (DH) model assumes that the light response curve (ETR vs. PPFD, also *J*:*I* curve) of photosynthetic electron transport (ETR‐LRC) can be represented by a combination of two intersecting rectangular hyperbolae with different limiting effects, hyperbola *H*1 interpolating data at low‐irradiance and hyperbola *H*2 interpolating data at high irradiance. The intercept (transition point) between the two hyperbolae usually falls at 100‐150 μmol photons m^‐^^2^ s^‐^^1^ in *P. vallarsae*. Both hyperbolae can be described by simple Michaelis Menten‐type equations:

$$\mathrm{ETR}=\left(V*I\right)/\left(K+I\right)$$

where *V* and *K* are the maximum velocity and the value of irradiance (*I*) when *ETR* = 0.5 V, respectively. The high‐light hyperbola *H*2 is defined by parameters *V*2 and *K*2, the low‐light hyperbola *H*1 is defined by *V*1 and *K*1. As a rule, *V*1>*V*2 and *K*1*>K*2. The two equations can be written as

ETR−H1=0

and

ETR−H2=0

and combined in a single equation of the second degree in ETR:

$$\left(\mathrm{ETR}-H1\right)*\left(\mathrm{ETR}-H2\right)=0$$

$${\mathrm{ETR}}^{2}-\left(H1+H2\right)*\mathrm{ETR}+\left(H1*H2\right)=0$$

whose lower root is

(eq.1) $$\mathrm{ETR}=\frac{\left(H1+H2\right)-\sqrt{{\left(H1+H2\right)}^{2}-4*H1*H2}}{2}$$

By substituting *H*1 with $\frac{V1*I}{K1+I}$ and *H*2 with $\frac{V2*I}{K2+I}$ the equation of the DH model is obtained:

(eq.2) $$\mathrm{ETR}=\frac{\left(\frac{V1*I}{K1+I}+\frac{V2*I}{K2+I}\right)-\sqrt{{\left(\frac{V1*I}{K1+I}+\frac{V2*I}{K2+I}\right)}^{2}-4\frac{\left(V1*I\right)\left(V2*I\right)}{\left(K1+I\right)\left(K2+I\right)}}}{2}$$

The abscissa of the intersection between *H*1 and *H*2 is:

(eq 3) $$I=\frac{V2*K1-V1*K2}{V1-V2}$$

**FIGURE 1:**
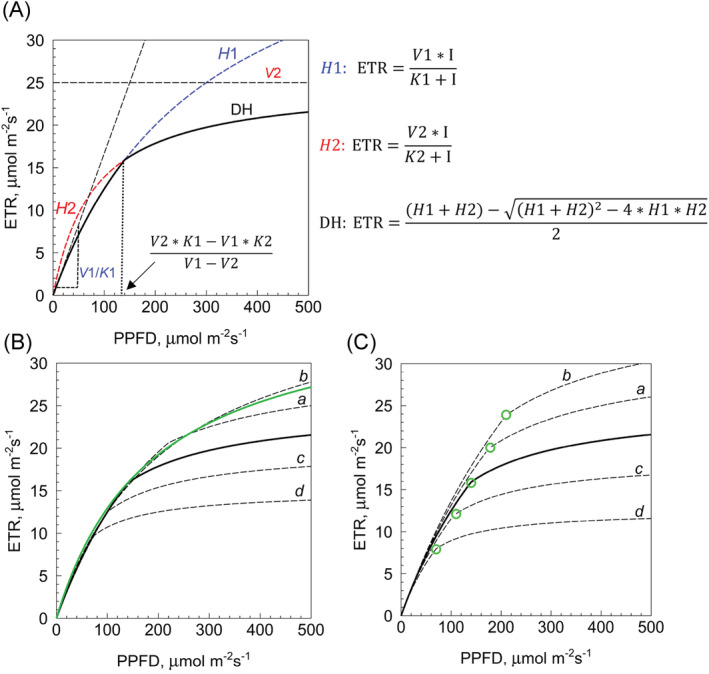
Graphical representation of the DH model and its main features. (A) The DH equation describes a discontinuous curve formed by hyperbolae *H*1 (dashed blue line) at low irradiance, and *H*2 (dashed red line) at high irradiance. The PPFD at which the two hyperbolae intersect can be derived from four parameters *V*1, *K*1, *V*2, *K*2 (eq. 3 in Methods). In this example, *V*1 = 50; *K*1 = 300; *V*2 = 25; *K*2 = 80 (V values in μmol electrons m‐^2^ s‐^1^, K values in μmol photons m‐^2^ s‐^1^), hence the intersection point occurs at PPFD = 140 μmol photons m^‐^^2^ s^‐^^1^. Other important features of the model include the asymptote ETR = *V*2 (corresponding to ETR_max_) and the *V*1*/K*1 ratio, maximum quantum yield of linear electron transport. (B) Bending of the DH curve becomes more marked by increasing the difference between *H*1 and *H*2 parameters, as shown by dashed curves *c (V*1 = 55; *K*1 = 340; *V*2 = 20; *K*2 = 60) and *d* (*V*1 = 60; *K*1 = 380; *V*2 = 15; *K*2 = 40) compared to the thick black curve (same of panel A). Vice‐versa, the discontinuity becomes smoother by decreasing the difference between *H*1 and *H*2 parameters, like in curve *a* (*V*1 = 45; *K*1 = 260; *V*2 = 30; K2 = 100) and curve *b* (*V*1 = 40; *K*1 = 220; *V*2 = 35; *K*2 = 120). The latter has the appearance of a rectangular hyperbola as the intercept lies at 1280 μmol photons m^‐^^2^ s^‐^^1^, well off the light range of our measurements. The green curve is the limiting rectangular hyperbola obtained when the *H*1 and *H*2 parameters are identical (here, *V*1=*V*2=37.5; *K*1*=K*2=190). (C) The intercept points of the DH curves (green circles) shift toward lower PPFD values when all *H*1 and *H*2 parameters decrease by a factor of 0.75 (curve *c*) or 0.5 (curve *d*); vice‐versa, the intercept moves toward higher PPFD values when *H*1 and *H*2 parameters increase by a factor of 1.25 (curve *a*) or 1.5 (curve *b*). In these examples, the ratios *V*1*/K*1 and *V*2*/K*2 are kept constant, hence the initial slope of all curves (*V*1*/K*1) is also constant.

The two hyperbolic functions describe two markedly different regions of an LRC, one at low and one at high irradiance, and their product is very effective in fitting experimental data. As shown in Figure 1 and Figure 3, a first hyperbola (*H*1) carefully describes the experimental data at low PPFD values, to be replaced by a second one (*H*2) at higher PPFD values when a different step becomes limiting, with an intercept in between. The effect on the light curve of varying *V* and *K* parameters, culminating into a rectangular hyperbola when *V*1 *= V*2, *K*1 *= K*2, is illustrated in Figure 1. Notably, the intercept point shifts to higher PPFD with increasing values of the major DH parameters.

While the hyperbolic behavior of ETR in high light (*H*2) is consistent with the kinetics of an enzyme whose substrate concentration is increasing with PPFD, the empirical evidence for a curvilinear fitting of LRC data at low irradiance (*H*1) points to an electron transport‐related enzymatic function of chloroplasts. Whilst *H*1 fitting is always perfect in *P. vallarsae*, the hyperbolic nature of the LI response may not be as obvious in other species (pseudo‐linear responses), particularly when ETR values are high. In a quite different approach, the biophysical model of Ye et al. (2013) assumes that the ETR response to increasing *I* is not linear due to a growing population of Chl excited states, with a consequent decrease of the population of ground state Chl. Ye's equation [ETR = α*I* (1*‐βI*)/(1+ γ*I*)] takes the form of a rectangular hyperbola (with *V* = α/γ and *K* = 1/γ) if the inhibition factor β is neglected. Moreover, Ye's function becomes indistinguishable from a rectangular hyperbola at low *I*, when the term (1‐β*I*) is negligible. Therefore, the use of hyperbolic function *H*1 to track ETR trends in low light is justified both on empirical and theoretical grounds.

Since a rectangular hyperbola is represented as linear in double‐reciprocal plots (ETR^‐^^1^/PPFD^‐^^1^), an LRC comprised of two rectangular hyperbolae takes a broken, hockey‐stick shape. The DH equation was used in most of the present work.

### 2.5 Statistical analysis

The data shown in the figures are means and SE of n repetitions, with n varying between 15 and 25 depending on the experiment as reported in the legends of the figures. If not otherwise stated, repetitions correspond to individual leaves of different plants. Statistically significant differences were evaluated by T‐test (p < 0.05 or less) or ANOVA with LSD test (significance 0.05).

## 3 RESULTS

### 3.1 Initial responses to water stress

**FIGURE 2:**
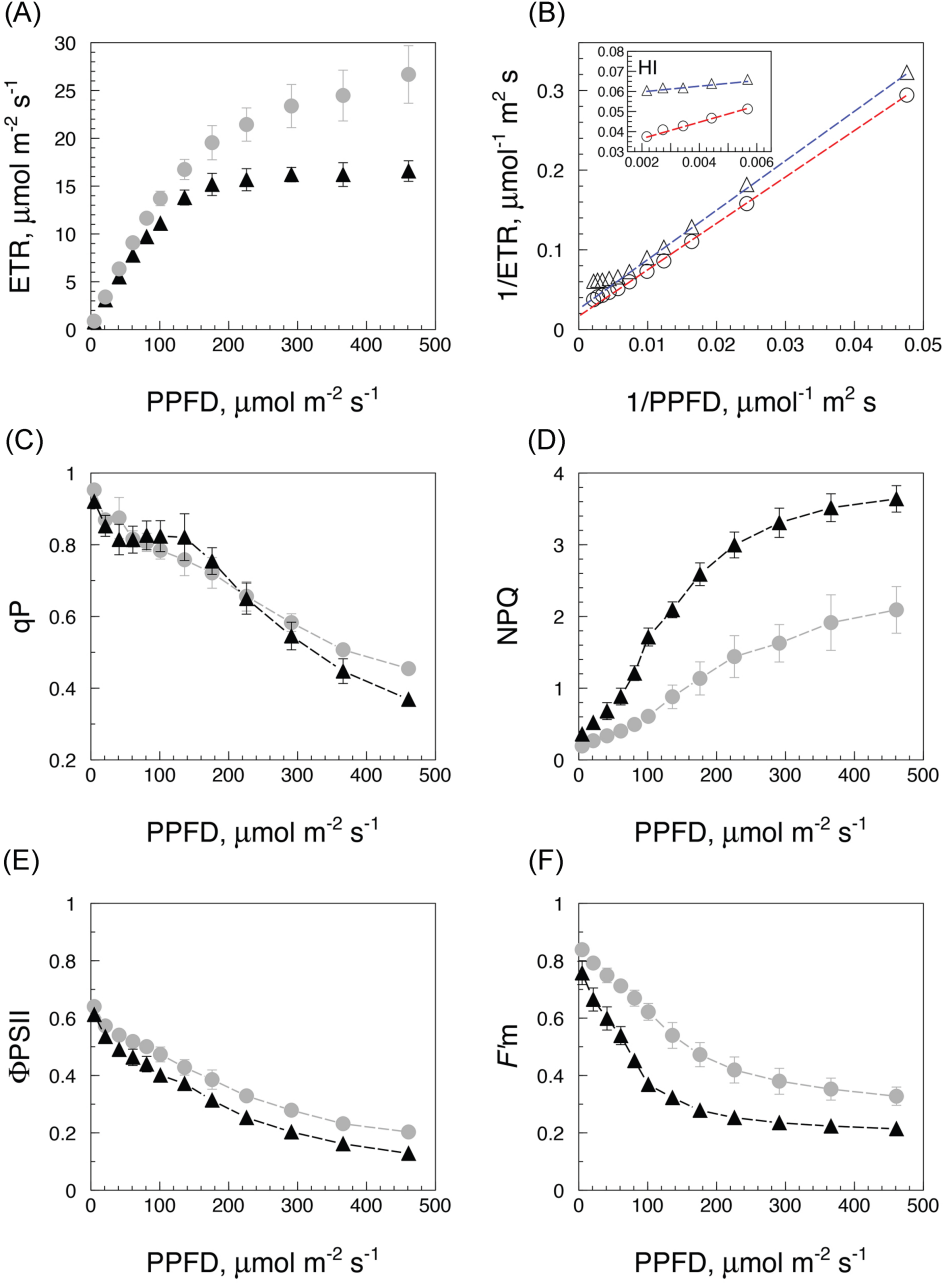
Light response curves (ETR‐LRCs) of three *P. vallarsae* plants (one leaf each) at the second day without watering, mean with SE. (A) At 10 am (*Dd*2, grey dots) and 11 pm (*Dn*2, black triangles). (B) Same data expressed as double‐reciprocal plot highlight the different slopes (*K/V*) of the LRCs in the low‐irradiance (LI) and high‐irradiance (HI) regions. The inset shows diverging LRCs at high irradiance due to a night BIC. Dynamics of photosynthesis‐related parameters during the LRC tests: qP (C), NPQ (D), *F*’_m_ (E), Φ_PSII_ (F).

Light response curves of photosynthetic electron transport (ETR‐LRCs) were investigated in leaves of water‐sufficient and water‐stressed *Pulmonaria vallarsae* by PAM fluorometry. Each LRC was preceded by a 5 min blue light induction pretreatment yielding values of several parameters (Φ_PSII_, qP, fluorescence peak FIP, NPQ). The ETR‐LRC of a slightly stressed plant (second day of water shortage) has the shape of a non‐rectangular hyperbola, somewhat flattened by night (Figure 2A). In a double‐reciprocal plot (Figure 2B), the LRCs take a hockey‐stick shape with an inflection point at 100‐150 μmol photons m^‐^^2^ s^‐^^1^ PPFD dividing two linear regions: a low irradiance (LI) domain (<100 μmol photons m^‐^^2^ s^‐^^1^) and a high‐irradiance (HI) domain (>100‐150 μmol photons m^‐^^2^ s^‐^^1^). The inset of Figure 2B highlights a divergence of morning and night LRCs at high PPFD due to an upward bend of the night response, reflecting a lowered night activity and a flattened LRC in the HI range, in contrast to nearly parallel trajectories at lower PPFD values. This type of effect is defined here as the bright illumination constraint (BIC) and is of frequent occurrence in water‐stressed plants mainly during night tests. A BIC is readily reversible by irrigation or daytime light (not shown) and might be a consequence of stomatal limitation, as discussed below.

The dynamics of photosynthetic ETR in an LRC are accompanied by modifications of several fluorescence‐related parameters. Photochemical quenching (qP) decreased slowly with increasing irradiance with slight differences between day (higher) and night (lower) trajectories, except for a clear night “hump”. The hump is typical of water‐stressed plants (Figure 2C) when the nocturnal course of qP twice crosses over the daytime course in the PPFD range of 60‐150 μmol photons m‐^2^ s‐^1^, suggesting a permanence of active reaction centres at medium‐low irradiance under stress conditions, concomitant to an NPQ rise. In fact, nonphotochemical quenching (NPQ) slowly increased with irradiance during diurnal tests, whereas the nocturnal NPQ response was stronger and much reinforced in slightly stressed leaves (Figure 2D). The effective yield of photosystem II (Φ_PSII_, Figure 2E) and the maximum relative fluorescence (*F*’_m_, Figure 2F) after an initial drop underwent a further, asymptotic decline with increasing irradiance, steeper during night tests (*F*’_m_ in particular).

### 3.2 Double‐hyperbola model

**FIGURE 3:**
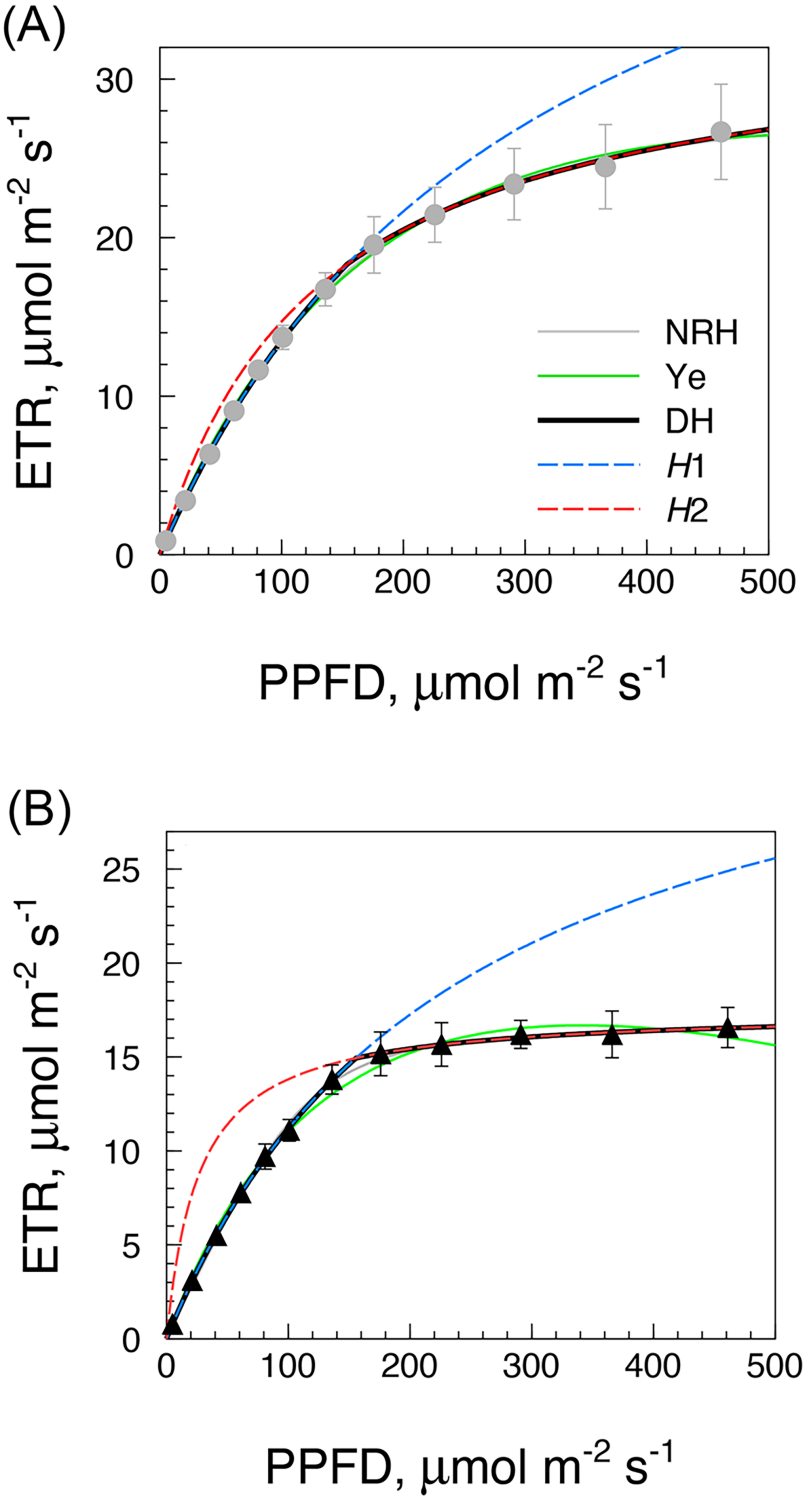
Comparison of alternative models in interpreting LRC data. Three different model equations were fitted to the LRC data of Figure 2: (A) *Dd*2 (day), (B) *Dn*2 (night). NRH (grey): Farquhar et al., 1980; Ye (green): Ye et al., 2013; DH (black): double‐hyperbola model. Dashed are the two virtual rectangular hyperbolae of DH model, i.e. *H*1 (LI domain, high *V*1; blue) and *H*2 (HI domain, *V*2 = ETR_max_; red).

In consideration of the variable and environment sensitive responses of the ETR‐LRC at high irradiance (HI) in contrast with more conservative trends in the LI range (Figure 2), we developed a method which considers the HI and LI regions of an LRC in terms of two separate but interacting rectangular hyperbolae. This approach results in a flexible model of the LRC based on the double‐hyperbola (DH) equation (see Methods and Figure 1). The robust fitting performance of the DH model applied to both ETR‐LRCs of Figure 2 is illustrated in Figure 3, in comparison with the NRH equation (Farquhar et al., 1980) and Ye model (Ye et al., 2013).

All three methods do fit the LRC data, although the equation of Ye et al. (2013) is slightly less performing in terms of r^2^ probably because its β term implies a declining trend of the curve at high illumination that is never seen in the irradiance range used in this work (see Figure 2D). The parameters obtained from the three fittings are reported in Table 1, which shows similar values of parameters Φ (NRH) or α (Ye) in both day and night LRCs. A remarkable stability also characterizes the *V*1*/K*1 ratio of the DH model since *V*1 and *K*1 usually vary in concert within and between experiments, while the *V*2/*K*2 relationship in the HI region can widely vary. Summarizing, the DH model compares favorably with other LRC models, with r^2^ = 1 in most applications (Table 1), and is most useful in case of nocturnal or stress responses. Consequently, the DH model was adopted in the larger study of photosynthetic adaptation to mild water deficit to be described.

**TABLE 1:** A comparison of alternative approaches to LRC fitting in the experiment of Figure 2, with 4 LRC tests: parameters and values yielded by three different equations (DH model, this paper; NRH model, Farquhar et al., 1980; Ye model, Ye et al., 2013)

Model	Parameter	Day values	Night values	units
*Double‐hyperbola (DH) model*				
	*V*1	55.1	37.7	μmol m^‐^^2^ s^‐^^1^
	*K*1	309	237	μmol m^‐^^2^ s^‐^^1^
	*V*2	33.8	17.5	μmol m^‐^^2^ s^‐^^1^
	*K*2	130	26.7	μmol m^‐^^2^ s^‐^^1^
	*V*1*/K*1	0.178	0.159	electrons/photons
	R^2^	0.999	0.995	
*Non‐rectangular hyperbola (NRH) model*				
	Φ	0.172	0.135	μmol(electrons) μmol(photons)^‐^^1^
	J_max_	30.6	17.1	μmol m^‐^^2^ s^‐^^1^
	θ	0.658	0.912	‐
	R^2^	1.02	0.994	
*Ye model*				
	α	0.193	0.177	μmol (electrons) μmol(photons)^‐^^1^
	β	0.000449	0.000809	μmol (electrons) μmol(photons)^‐^^1^
	ϒ	0.00366	0.00475	μmol (electrons) μmol(photons)^‐^^1^
	R^2^	0.965	0.979	

### 3.3 Photosynthesis under incipient drought

**FIGURE 4:**
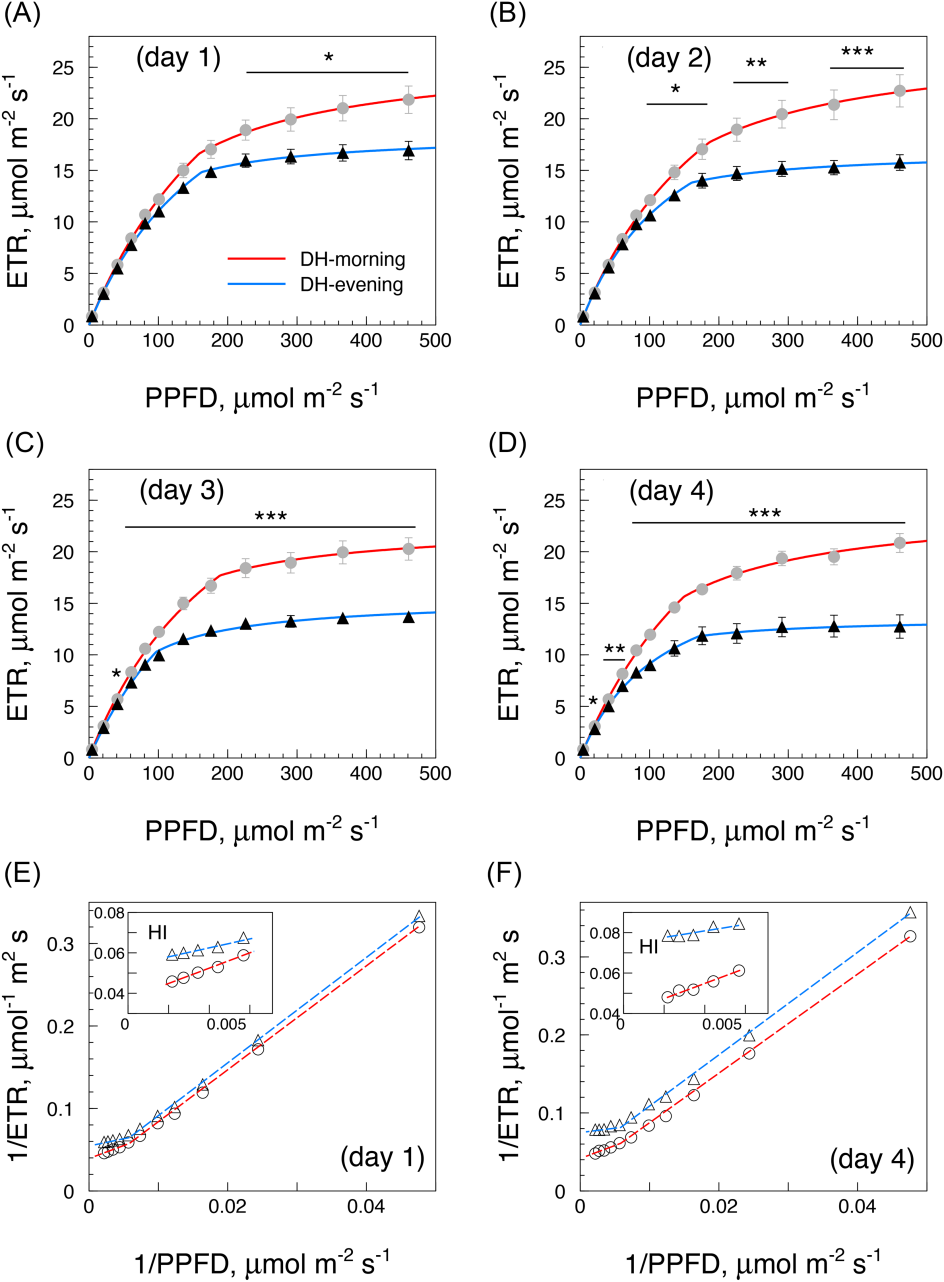
*P. vallarsae* light response curves under increasing water deficit, means of 25 experiments of four‐days’ water restriction (152 LRC tests). (A) to (D) (days 1 to 4) report mean values and SE of 19 ETR‐LRCs performed on average in each condition, one LRC test being made in the morning (ca. 10 am, grey dots) and one in the night (11 pm, black triangles) for four days, using one leaf for each experiment. Statistical differences (T‐test) between morning and night values: * p < 0.05; ** p < 0.01; *** p < 0.001. (E) and (F): double‐reciprocal plots of the data of day 1 (*Dd*1, *Dn*1) and day 4 (*Dd*4, *Dn*4) respectively, showing BIC effects in the HI region (insets).

**FIGURE 5:**
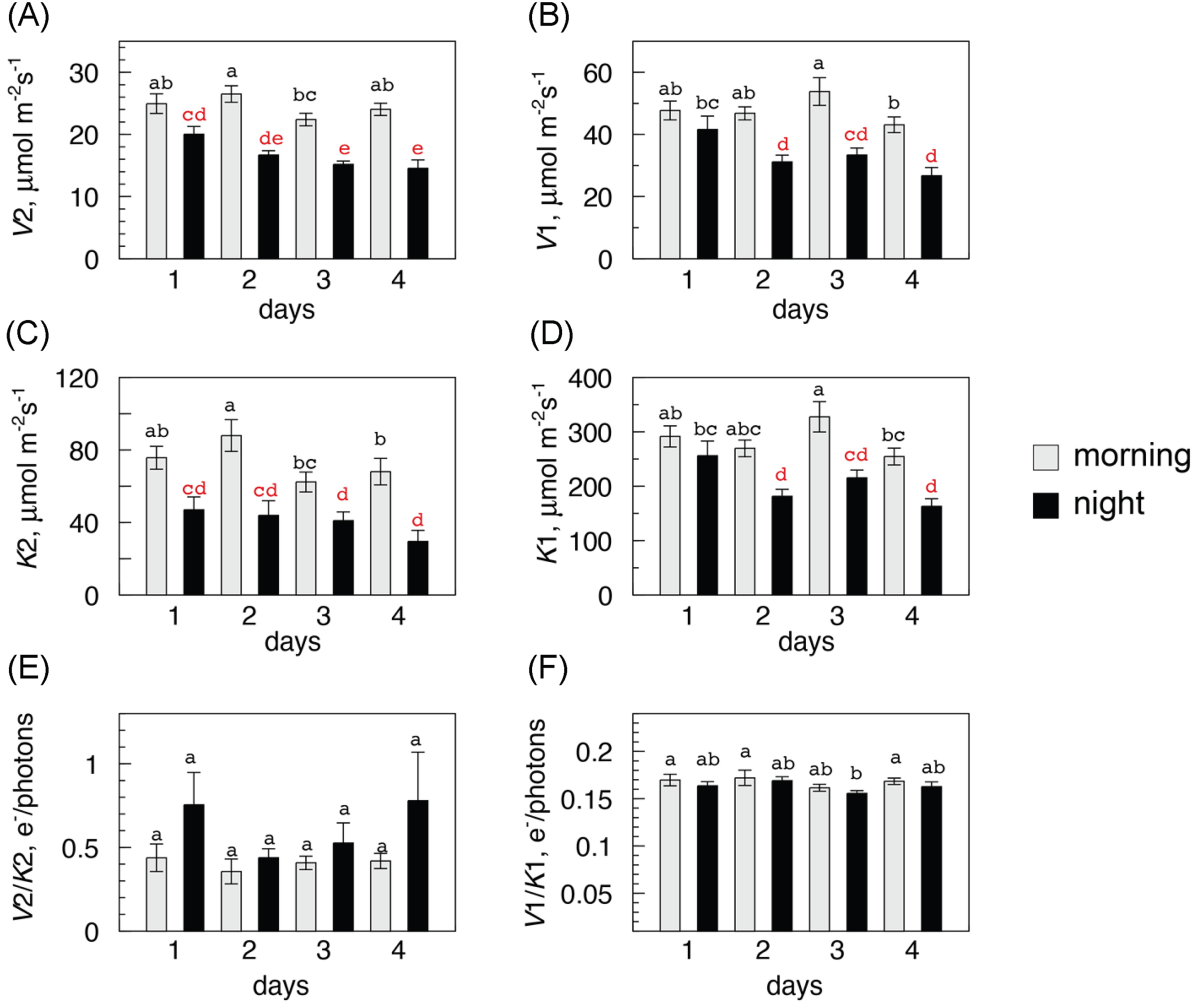
The DH model parameters in the experiments of Figure 4. (A) to (D): trends of ETR‐LRC parameters (*V*1, *K*1, *V*2, *K*2; means with SE) during four days of water restriction, calculated using the DH equation and reported as bar diagrams (grey = morning, black = night). Statistical analysis was performed with ANOVA (LSD test, significance 0.05), and significant differences are highlighted by different letters. Red letters pinpoint those nocturnal values that are significantly different from the morning values of the same day. E), F): *V/K* functions showing fairly constant *V*2*/K*2 (though with large variance) and constant *V*1*/K*1 ratios.

The main results of 25 experiments of 4 days’ water deprivation in *P. vallarsae*, always using one leaf per plant, are summarised in Figs. 4, 5. The mean results of two ETR‐LRC tests a day repeated for four days, morning and night, are presented in Figure 4 (A to D). Some differences between daytime and nighttime photosynthetic responses were already noticeable at the start (*Dd*1 vs. *Dn*1, Figure 4A), and the day/night gap continued to widen during three further days of water restriction due to a progressive flattening of the night curves (Figure 4 B‐C‐D). Figure 4 E‐F shows data for the first and the last (fourth) day in the form of double‐reciprocal plots, exhibiting linear and parallel trajectories of the LRCs by day and night with an inflection around 100‐150 μmol photons m‐^2^ s‐^1^ PPFD. The insets emphasize BIC‐like deviations of nocturnal responses at high irradiance.

**FIGURE 6:**
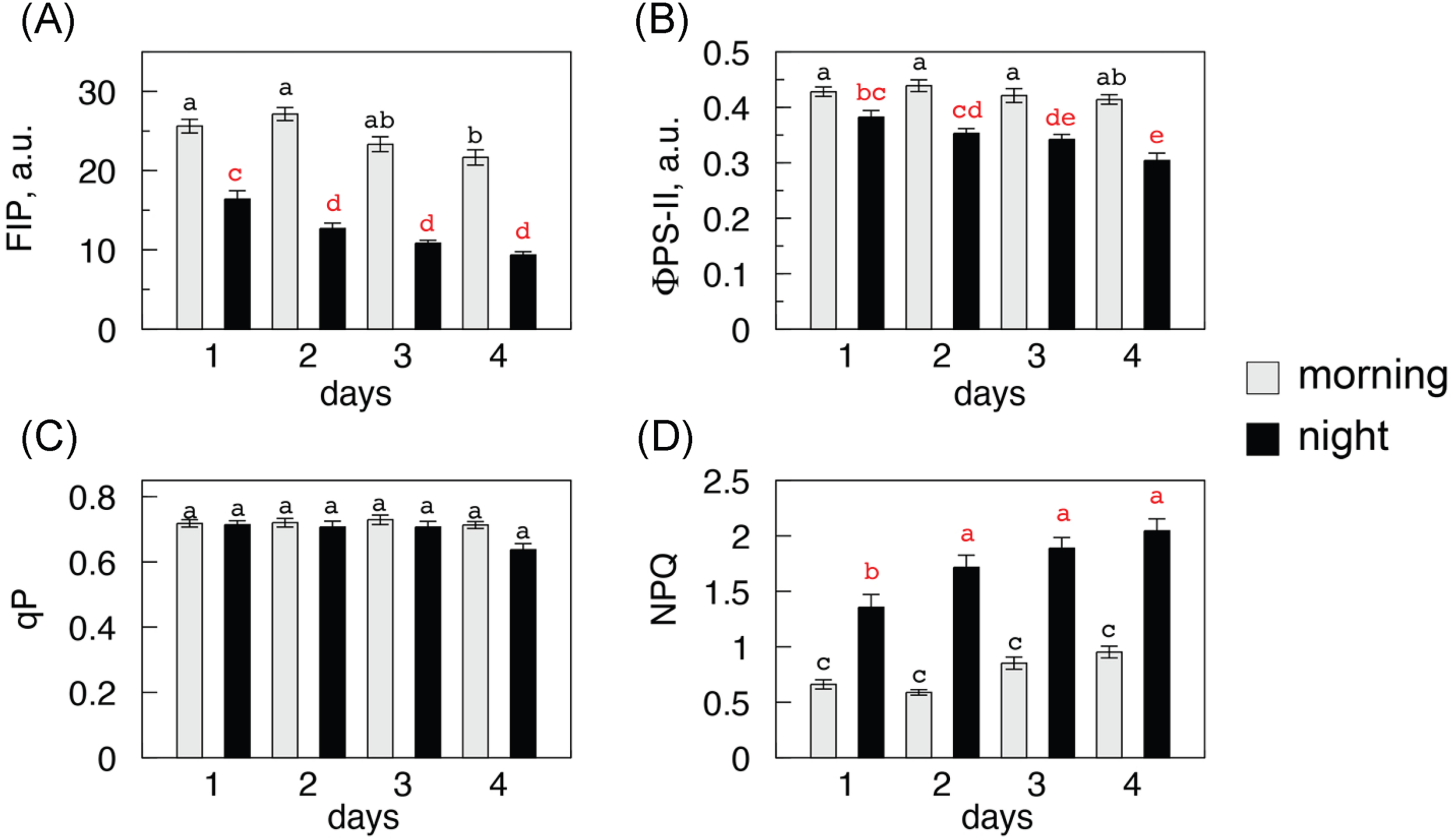
Trends of fluorescence‐related parameters in 25 water stress experiments (Figs. 4, 5): FIP (A), Φ_PSII_ (B), qP (C), and NPQ (D). Grey = morning, black = night. Mean values and SE of data recorded at the end of light activation treatment preceding each LRC. Statistical analysis performed with ANOVA (see Figure 5).

The whole process is further illustrated in Figure 5 (A to D), where bar diagrams document the decline of nocturnal values of *V*1, *V*2, *K*1, and *K*2 during four days of water shortage, while diurnal values remained relatively stable. The nocturnal values in the HI domain (*H*2 curve) started to decline already during the first night, while the nocturnal *H*1 values were significantly lower starting from the second night on. This dual day/night trend underscores the specific and growing impact of the night depression of photosynthesis during a continued water deficit stress. During the 4‐days’ experiment, the maximum quantum yield of photosynthesis (*V*1/*K*1) calculated over all data (Table 1) was fairly constant, no matter whether the measures were performed by morning (0.178 ± 0.005 e^‐^/photon, n = 4) or by night (0.159 ± 0.007 e^‐^/photon, n = 4). Night LRCs were also characterized by intercepts at lower PPFD values than daytime curves (124 ± 19 vs. 151 ± 18 μmol photons m^‐^^2^ s^‐^^1^), indicating that the LI range was significantly enlarged during the day as anticipated by the DH model.

The analysis was extended to other fluorescence related parameters (Figure 6, A to D) whose values were measured at the end of the blue light induction pretreatment preceding LRC tests and averaged over 25 experiments. Each parameter gave a specific response. Nocturnal FIPs were low at the beginning and rapidly dropped in successive nights, but less rapidly by day; Φ_PSII_ was stable by day though slowly decreasing in the night; qP remained constant by day and night until the end of the experiment. By contrast, NPQ rose asymptotically one night after another, with a small daytime increase. In summary, most photosynthesis‐related parameters except qP and NPQ were stable or underwent minor changes during the day throughout the experiments, while their night values were significantly decreased. After four days of water restriction, the trials were stopped as most plants showed a drop of daytime photosynthesis to “dark” levels, often accompanied by visible wilting. The previous state of photosynthetic activity was recovered within 3‐4 days upon irrigation (data not shown).

### 3.4 Photosynthesis at low water stress

**FIGURE 7:**
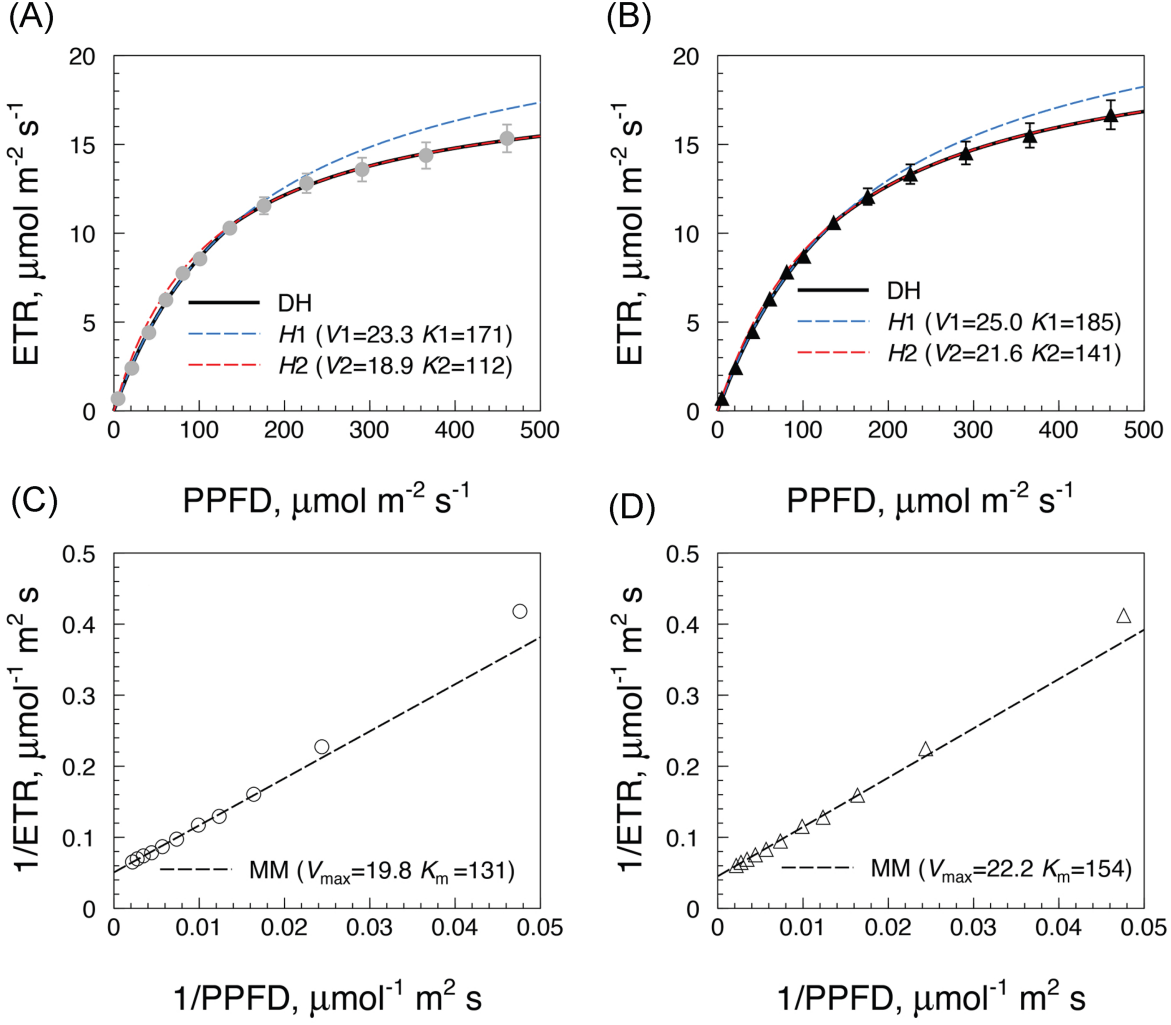
A winter ETR‐LRC experiment with *P. vallarsae*. A single leaf for each of three potted plants kept outdoor was tested two‐times a day (ca. 10 am and 10 pm) at 3‐days intervals from January 24 to February 5, 2024. (A), (B) The two LRCs represent mean data and SE of 15 morning tests (dots) and 15 evening tests (triangles), fitting by DH equation. The *H*1 and *H*2 hyperbolae and respective *V* and *K* parameters are highlighted. (C), (D) Same data as double‐reciprocal plots, the straight dashed lines being drawn by data fitting with the Michaelis‐Menten (MM) equation; parameters *V*_max_ and *K*_m_ are comparable to *V*2 and *K*2. Units of parameters in the figure are μmol electrons m^‐^^2^ s^‐^^1^ (*V*1, *V*2, *V*_max_) and μmol photons m^‐^^2^ s^‐^^1^ (*K*1, *K*2, *K*_m_).

For comparison with the above experiments conducted in late spring and summer, we also investigated midwinter responses of outdoor growing *P. vallarsae*. In contrast to summer responses, winter LRCs of the leaves were similar during day and night, with no trace of night depression (Figure 7). In fact, most LRCs were slightly higher by night, possibly a consequence of diurnal photoinhibition. The shape of the LRC approximated a rectangular hyperbola, although two slightly diverging hyperbolae could be detected by data fitting with the DH model. By fitting with the Michaelis‐Menten equation, the result resembled that of the *H*2 equation (Figure 7). Therefore, the DH model can be applied to all growth conditions with or without stress in this species.

## 4 DISCUSSION

### 4.1 Circadian rhythm and drought

The rhythmic modulation of plant photosynthesis and many of its individual components has been long known to be under the control of a circadian oscillator, finely tuned to the seasonal light‐dark regime (Dodd et al., 2014; Millar, 2016). Circadian oscillations of Chl fluorescence have been reported in many plants, including liverworts (Gould et al., 2009; Litthauer et al., 2015; de Barros Dantas et al., 2023). Pupillo et al., (2022) using pulse amplitude modulated (PAM) fluorometry investigated the circadian night depression of photosynthesis in *Pulmonaria vallarsae*, a lungwort species known for its continued photosynthesis through most of the year (Recchia et al., 2017). The night effect was apparently associated to the experience of a moderate water stress (Pupillo et al., 2022) and was largely dependent on reduced rates of nocturnal photosynthetic electron transport capacity and fluorescence yield, concomitant with an NPQ outburst (Bassi & Dall’Osto, 2021). Given the ample evidence of coordinated regulation of photosynthetic activity and stomatal movements (Pallas et al., 1974; Wong et al., 1979: Kerr et al., 1985; Sakoda et al., 2021), the circadian rhythm of photosynthesis can be envisioned as a major component of a complex syndrome basically aimed at optimal water use control. However, the rhythm also opens the way to “dark” cell activities thanks to concurrent changes of redox functions, enzyme activities and other cell conditions (e.g., chloroplast oxidative functions and altered pyridine nucleotide balance: Trost et al., 1993; Cejudo et al., 2019; Yokochi et al., 2021; Gurrieri et al., 2021, 2023). Of course, circadian rhythms regulate a large variety of physiological processes (possibly a majority) in addition to photosynthesis related functions, in a whole‐plant crosstalk (Millar 2016).

### 4.2 Plants and water deficit stress

Plants responses to water deficit stress include the enhancement of several functions regulated by circadian rhythms. Stressful conditions are thought to select for robustness of circadian rhythms in plant populations (Yerushalmi et al., 2011) resulting in improved fitness (Dodd et al., 2005; Dakhiya et al., 2017). The nocturnal closure of stomata, a circadian process itself (Martin & Meidner, 1971), is known to occur under water stress in many species, and stomatal regulation may be weakly or not expressed in well‐irrigated plants (Raschke, 1975; Mansfield et al., 1990; Caird et al., 2007; Lawson et al., 2010). As shown in this paper, *P. vallarsae* plants exposed to the inclemency of winter weather, hence with little or no experience of water deficit stress for months, exhibit similar photosynthetic responses by day and night.

Another important contribution to drought tolerance in plants is afforded by the stress‐ and rhythm‐dependent control of hydraulic conductance by aquaporins (Flexas et al., 2008; Li et al., 2014; Kapilan et al., 2018), that is turned off upon drought relief (Caldeira et al., 2014). The present study of the effects of four days of water shortage in *P. vallarsae* sheds some light on the circadian photosynthetic response, that was also addressed in a proteomic perspective in Arabidopsis (Greenham et al., 2017). An early marker of the stress adaptation process, mainly during night tests, was a characteristic flattening of the ETR‐LRC at relatively high illumination, here defined as the bright illumination constraint (BIC). By analogy with examples of LRCs at low ambient CO_2_ (e.g. von Caemmerer 2000), a BIC can be understood in terms of CO_2_ limitation following partial stomata closure and may represent an early, reversible step of physiological adaptation to water shortage. Since the total internal CO_2_ pool of *Pulmonaria* leaves may not exceed 10 μmol m^‐^^2^, it can be safely concluded that any photosynthetic activity would rapidly deplete the intercellular spaces of CO_2_ in the absence of efficient stomatal gas exchange. Further progress in the stress adaptation process resulted in progressive distancing of day and night LRCs as the outcome of a continued decline of nocturnal photosynthetic capacity, with a concurrent increase of NPQ. But while diverging, morning and night ETR‐LRCs maintain a stable relationship as evidenced by their similar *V*1*/K*1 ratios (in terms of the DH model, this paper), and consequent co‐linearity of the LI (*H*1) responses in double‐reciprocal plots (Figure 4E‐F; see Pupillo et al., 2022).

The decline of photosynthetic capacity in water stressed plants, with accompanying stomatal regulation (Graan & Boyer, 1990; Gunasekera & Berkowitz, 1993; Tezara et al., 2002), is consistent with the abundant evidence of oxidation of redox proteins and metabolic inhibition during water deficit (Daszkowska‐Golec & Szareiko, 2013; Noctor et al., 2014; Zaffagnini et al., 2019), with a gradual transition to alternative electron sinks and cyclic electron transport (Zivcak et al., 2013). That severe drought can heavily damage the photosystems is now well established (Hu et al., 2023; Sapeta et al., 2023), but such effects may not extend to less stressful conditions (Chen et al., 2016). It was also argued that mild water stress does not impair photosynthesis, the decline of which was entirely attributed to stomatal restriction of CO_2_ flux (Mansfield et al., 1990; Cornic & Briantais, 1991; Bota et al., 2004) since the activity was apparently restored by saturating levels of CO_2_ (Chaves et al., 2009), but the issue remains unsettled (Lawlor & Tezara, 2009). Experimental conditions, especially stress levels, may be decisive here, and species responses also differ (Quick et al., 1992; Castrillo et al., 2001; Parry et al., 2002). Although increased leaf mesophyll resistance to CO_2_ diffusion has been suggested to contribute to the decline of photosynthesis during water shortage (Flexas et al., 2008), the fact that the intercellular CO_2_ partial pressure in water‐stressed leaves was elevated or even augmented (Forseth & Ehleringer, 1983; Gunasekera & Berkowitz, 1993; Lal et al., 1996; Sanchez‐Rodriguez et al., 1999) is difficult to reconcile with a solely stomatal limitation.

The present results with *P. vallarsae* support a nocturnal, stress‐dependent, inhibition of photosynthetic metabolism under circadian control (Pupillo et al., 2022) and, consequently, of photosynthetic electron transport by a feedback loop. The inhibitory effect known as night depression of photosynthesis is characterized by robustness and stability well beyond the night decline of potential photosynthesis commonly observed during a natural light/dark cycle in water‐sufficient plants, as demonstrated by its complete insensitivity to activation treatments (including the blue light pulse induction which precedes any LRC test). The night depression effect is apparently associated with an increasing biochemical inhibition of C3 cycle enzymes and Rubisco (and Rubisco activase: Yamori et al., 2012), mainly as a consequence of a changed redox environment (Cejudo et al., 2019; Gurrieri et al., 2021, 2023), and with concomitant stomatal movements, as also suggested by BIC effects. Similar conclusions were drawn for (diurnal) photosynthesis in several plant species subjected to moderate water stress (Wong et al., 1979; Forseth and Ehleringer 1983; Martin & Ruiz‐Torres, 1992; Tezara et al., 2002). Lauer and Boyer (1992) argued that “the onset of water deficit … caused simultaneous reductions in stomatal apertures and biochemical capacity for photosynthesis, but biochemical capacity limited photosynthesis and stomatal closure did not”. This concept fits well with our experiments, those under nocturnal (and entirely artificial) photosynthesis conditions in particular. The dependence of stomatal movements on mesophyll driven signals is currently investigated (Lawson et al., 2014; Lawson & Matthews, 2020).

### 4.3 The light response curve and the double‐hyperbola (DH) model

The much‐supported assumption that the low‐irradiance (LI) region of an LRC is conditioned by the RuBP regeneration phase of C3 cycle, strictly dependent on ATP/NADPH supply, whereas the high‐irradiance (HI) region is Rubisco limited (see von Caemmerer, 2000), suggests itself separate treatments of the two major domains. Upon inspection, both domains show a hyperbola‐like shape that may recall the partial saturation curves of two different, limiting enzyme systems. In contrast to the LI domain, the HI domain of an LRC appears to be readily sensitive to diverse environmental effects, resulting in highly variable responses as expressed by BICs. Thus, there were good grounds to test a mathematical treatment of the LI and HI domains as distinct but interacting components of the LRC. The double‐hyperbola (DH) model thus constructed is based on four parameters (*V*1 and *K*1, *V*2 and *K*2) representing maximum rate and light dependence of photosynthesis in the LI and HI domains, respectively, as expressed by *H*1 and *H*2 hyperbolae. This allows a direct biochemical reading of the curve through its major parameters. In this respect, established LRC models have some shortcomings, e.g. most NRH models include a curvature parameter (θ) whose biological meaning is unclear. As to the recent theory of Ye (Ye et al., 2013, 2024) based on the excitation states of chlorophylls, the LI domain is viewed as hyperbolic while the HI domain features an ETR maximum after which the curve starts declining. This does not occur in *P. vallarsae* at the maximal irradiances tested. Also, the metabolic reactions of photosynthesis are only taken into account through a rate term (ξ_2_ *R*_1_) included in the definition of β and γ parameters.

A basic requirement for the DH model was its reliability in terms of data fitting, and this condition was satisfactorily fulfilled when the model was tested on real LRCs. In fact, the product of two rectangular hyperbolae resulting in a non‐rectangular hyperbola affords a very good fitting to the experimental data (Figure 2). The first hyperbola (*H*1) accurately describes the trend at low PPFD values, to be replaced by a second one (*H*2) at higher PPFD when a different, metabolic step becomes limiting. In our work, the extrapolated, “native” strength of the electron transport rate *V*1 (LI domain) usually exceeded *V*2 (HI domain) by about 2‐fold on average, but this ratio was rather variable depending on *H*2 limitations, BIC effects, and possible errors in *V*1 and *K*1 extrapolation. Besides changes of rate (*V*1 and *V*2) and light requirements (*K*1 and *K*2), other minor oscillations of the LRCs during water shortage experiments are highlighted by DH model fittings, for example the periodic shifts of the *H*1*‐H*2 intercepts outlined in Results.

Examples of discrepant dynamics of the two major domains of an LRC can be found in the literature (von Caemmerer, 2000). An interesting case is the light response of *Eucalyptus* leaves as influenced by external CO_2_ (Ögren & Evans, 1993) when an increase of pCO_2_ from 35 to 100 Pa enabled the LI domain to engulf the HI domain, thus giving rise to a perfect rectangular hyperbola. In fact, our *H*1 curve under some conditions can extend to cover the whole light energy range of an LRC, which then takes the form of a single rectangular hyperbola (Figure 1), as regularly observed in *P. vallarsae* during winter months (Figure 7). In conclusion, the present work shows how common photosynthetic LRCs showing distinct low‐ and high‐irradiance regions can be reshaped by environmental conditions, with a more flattened HI response and a shortened LI region by night and, probably, at any time CO_2_ runs short, and vice‐versa, a hyperbola‐like course without gross discontinuity under less stressful conditions. These situations are well described by the flexible DH equation, ready to pinpoint subtle changes of LRC responses with possible insights into the physiological events within the leaf.

## AUTHOR CONTRIBUTIONS

P.P. and P.T. conceived the work; P.P. performed the experiments; B.A.M. and P.T. developed the DH model; P.T. performed the data analysis; P.P. wrote the manuscript; P.P., B.A.M., F.S., and P.T. critically reviewed and edited the manuscript. All authors contributed to the paper and approved the final version of the manuscript.

## FUNDING

This research did not receive any specific grant from funding agencies in the public, commercial, or not‐for‐profit sectors.

## Data Availability

Data sharing is not applicable to this article as all new created data is already contained within this article.
